# Associated factors of depression in pregnant women in Korea based on the 2019 Korean Community Health Survey: a cross-sectional study

**DOI:** 10.4069/kjwhn.2022.02.03.1

**Published:** 2022-03-17

**Authors:** Eun Gyeong Kim, Sook Kyoung Park, Ju-Hee Nho

**Affiliations:** 1 Department of Nursing, Kunsan National University, Gunsan, Korea; 2 College of Nursing, Research Institute of Nursing Science, Jeonbuk National University, Jeonju, Korea

**Keywords:** Depression, Health behavior, Health status, Pregnancy

## Abstract

**Purpose:**

Various individual and social factors influence depression in pregnant women. The purpose of this study was to identify the influence of socioeconomic status, health behaviors, and health status on depression of pregnant women in Korea.

**Methods:**

This study analyzed data from the 2019 Korean Community Health Survey conducted from August to October 2019. A structural questionnaire with Patient Health Quetsionnaire-9 (PHQ-9), health behavior, health status, and psychological characteristics was used. The data of 1,096 pregnant women between the ages of 19 and 55 years were analyzed using descriptive statistics, independent-test and chi-square tests, and multiple regression.

**Results:**

The mean score of prenatal depression as measured by the PHQ-9 during pregnancy was 2.35 points out of 0 to 27 points. Low income (B=0.69, *p*<.001), low-education level (B=0.70, *p*<.001), skipping breakfast (B=0.34, *p*=.001), less than 8 hours of sleeping (B=0.26, *p*=.009), binge drinking during pregnancy (B=0.46, *p*=.001), and stress (B=1.89, *p*<.001) were significantly associated with increased depression scores. In contrast, depression scores significantly decreased as subjective health status (B=–0.59, *p*<.001) and subjective oral health status (B=–.17, *p*=.003) increased.

**Conclusion:**

Findings support the need for healthcare policies and clinical screening to alleviate prenatal depression, especially for pregnant women with low socioeconomic status, poor health behavior, poor health status, and high stress.

## Introduction

Pregnancy carries a positive meaning as a normal developmental process that requires a woman’s physiological and psychological adaptation. Pregnant women are, nonetheless, vulnerable to a variety of mental health problems may have fears of giving birth or harbor feelings of inadequacy concerning their role as a mother, as well as experiencing mixed feelings and negative thoughts about pregnancy [1-3]. Among these considerations, depression is common among pregnant women, with 25.6% of expectant mothers worldwide experiencing the disease, and contributing to the emergence of a major public health problem [4-6]. In South Korea (hereafter Korea), the rate of depression experienced during pregnancy in 2008 was 26.5% [7], which increased to 41.0% in 2018 [8]—an increase of more than 15% over 10 years. In addition, the incidence of depression during pregnancy—including incidences of mild depression—was reported being as high as 35.9%, with reports suggesting that many Korean women experience moderate depression during pregnancy [9]. In light of these facts, identifying factors associated with depression as it appears during pregnancy is required.

Depression during pregnancy has been identified as a risk factor along with other major health problems that cause problems in fetal development, including premature births, a low birth weight, and the risk of adverse postnatal and cognitive-emotional function development in children [10-13]. In addition, depression may also manifest in children after childbirth [14]. Criticism of the family, or the transmission of depression to the husband [15,16], is known to have a negative impact on familial and social relationships.

Various individual factors have been reported as influencing the likelihood of depression during pregnancy, including a higher age, lower education level, no occupation, lower household income, and lower socioeconomic status [14,17-19]. Pregnancy-related characteristics, including the experience of a miscarriage and history of prepregnancy depression, have also been reported as factors that lead to an increased likelihood of prenatal depression [10,20]. It has also been reported that sleep disturbances, a health behavioral factor, affect prenatal depression [21]. In addition, some studies revealed that poor health status and higher perceived stress levels were associated with higher depression during pregnancy [9,19,22]. As hormonal changes lead to changes in oral conditions in pregnancy, oral health status has also been reported as related to depression [23].

However, in previous studies on factors related to depression in pregnant women, various factors were reported individually [14,17-20], and health behavior factors, were commonly omitted [21]. There are insufficient studies to comprehensively examine the effects of socioeconomic factors, health behaviors, health status, and psychological factors on depression. Therefore, this study aimed to examine the socioeconomic status, health behaviors, health status, and psychological factors of pregnant women in Korea, using nationally representative data, with the purpose of providing practical evidence-based data that can contribute to the prevention and control of depression during pregnancy.

## Methods

Ethics statement: Obtaining informed consent was exempted by the Institutional Review Board of Jeonbuk National University (2021-08-015) for this secondary analysis, because there was no sensitive information and the data were anonymously treated.

### Study design

This secondary analysis study used data collected from the 2019 Korea Community Health Survey (KCHS), employing a correlational survey design. The report adhered to the STROBE (Strengthening the Reporting of Observational Studies in Epidemiology) guidelines (https://strobe-statement.org/).

### Setting/data source

The KCHS is a nationwide health survey and sampling is selected through probability-proportional system extraction so that an average of 900 people can be sampled from 253 cities. It is designed to produce community-based health statistics at the city level, country level, and district units. From anonymized raw data approved from the 2019 KCHS, women who answered ‘pregnant’ to the question of ‘Are you between the ages of 19 to 55 years and currently menstruating?’ were selected. Non-response to questions related to study variables were excluded (n=1) and only complete data were used for final analysis. The data were collected from August 16 to October 31, 2019. KCHS employs trained data collectors and uses the computer-assisted personal interviewing method. Out of the 229,099 subjects who participated in the 2019 survey; data from 1,096 pregnant women were used for final analysis in this study.

### Measurements

#### Depression

Depression was measured using the Korean version of the Patient Health Questionnaire-9 (PHQ-9) [24]. The PHQ-9 is widely used as a screening tool for major depressive disorders and is known to have high degrees of sensitivity (88%) and specificity (88%) [22], and has been identified as appropriate for assessment in pregnant women [25]. Each of the nine items is scored on a 4 point Likert (not at all, 0 to nearly every day, 3), with higher total summed scores (range, 0–27; none, 0–4; mild, 5–9; moderate, 10–14; moderately severe, 15–19; severe depression, 20–27) indicating greater levels of depression [22]. At the time of development, the Cronbach’s α of the PHQ-9 was 0.84 [24]; and for this study, it was 0.79.

#### Health behaviors

Measured health behaviors included skipping breakfast, sleep duration, and binge drinking. For the item ‘How many days have you eaten breakfast in the past week?’, those who ate less than 5 times a week were classified as skipping breakfast. Regarding sleep duration, responses to the question ‘How many hours do you usually sleep in a day?’ were classified as either ‘8 hours or more’ or ‘less than 8 hours.’ Current binge drinking was defined as having answered ‘yes, more than once a week’ to the question ‘How often do you drink more than five glasses (or about three cans of beer) at a single drinking event?’

#### Health status

Health status included subjective health status, subjective oral health status, and unmet medical care. Subjective health and oral health levels were measured on a 5 point Likert (very poor, 1 to very good, 5), with higher average scores (possible range, 1–5) indicating better subjective health and/or oral health. Unmet healthcare needs were defined as ‘yes’ to the question ‘During the past year, did you not go to a hospital for care, despite wanting to?’

#### Psychological characteristics

Stress was measured as a psychological factor. The stress score is calculated by inversely converting the 4-point Likert score (quite a lot, 1 to very little, 4) to the question ‘How much stress do you usually feel in your daily life?’ A higher score indicated a higher level of stress.

#### Demographic characteristics

The general characteristics were age, household monthly income, and level of education. Household monthly income was reclassified into strata of 4 million and greater Korean won (KRW; approximately 3,600 US dollars) or less than 4 million KRW, per month. For educational level, the following options were available: ‘no formal education,’ ‘elementary school,’ ‘middle school,’ ‘high school,’ ‘2/3-year college,’ ‘4-year college,’ and ‘graduate school or higher’. Thereafter, the categories were simplified to ‘up to high school’ and ‘junior college graduate or more.’

### Data analysis

The data were analyzed using IBM SPSS Statistics ver. 21.0 (IBM Corp., Armonk, NY, USA); independent-test and chi-square tests were performed for differences relating to the participants’ characteristics. Cronbach’s α coefficient was calculated so as to verify the reliability of the instrument. Multiple regression analysis was subsequently performed to identify depression-related factors in pregnant women. Considering that the KCHS data is a complex sample design, individual weights were applied for accurate estimation.

## Results

### General characteristics and research variables of study participants

The mean age of the participants was 33.9 years. Forty-one percent of respondents reported a monthly household income of less than 4 million KRW. Only 8.1% self-identified as binge drinkers, while 49.4% of participants had skipped breakfast. The mean depression score was quite low at 2.35 points and 4.6% were identified with moderate or higher levels (score of 10–27) of depressive mood. Out of 1 to 5, the mean score of subjective health status was midpoint level (2.58) and slightly higher for oral health status (3.13). Women who experienced unmet healthcare needs accounted for 3.9% and the mean stress score was 1.98 out of 1 to 4, suggesting a roughly midpoint level of stress (Table 1).

### Comparison of depression scores by participants characteristics

Correlation analysis revealed a significant positive correlation of moderate strength between depression and stress (r=.42, *p*<.001). Furthermore, there were weak but significant negative correlations between depression and subjective health status (r=–.26, *p*<.001) and subjective oral health status (r=–.20, *p*<.001). Depression scores were significantly higher for households with a monthly household income of less than 4 million KRW (t=7.13, *p*<.001), for those with final education level of up to junior college (t=2.10, *p*=.037), women who skipped breakfast (t=8.62, *p*<.001), slept less than 8 hours (t=3.40, *p*=.001), engaged in binge drinking (t=4.43, *p*<.001), and had unmet healthcare needs (t=7.10, *p*<.001) (Table 1).

### Associated factors of prenatal depression

In terms of general characteristics, those with a reported monthly household income of less than 4 million KRW (B=0.69, *p*<.001) and, up to high school (B=0.70, *p*<.001) were more likely to be depressed. In health behaviors, depression scores were also more likely in women who skipped breakfast (B=0.34, *p*=.001), slept less than 8 hours (B=0.26, *p*=.009), and self-reported as a binge drinker (B=0.46, *p*<.001). In terms of health status, as scores for subjective health status (B=–0.59, *p*<.001) and subjective oral health (B=–0.17, *p*=.003) increased, the depression score decreased with statistical significance. As for the psychological characteristic, the higher the stress score, the higher the depression (B=1.89, *p*<.001). The explanatory power of these variables’ ability to explain depression in pregnant Korean women was 24.4%, with the model being deemed suitable (Wald F=268.61, *p*<.001) (Table 2).

## Discussion

The level of depression of this study is lower than a previous study in Korean pregnant women, which used the Edinburgh Postnatal Depression Scale (EPDS) [18]. Although it is difficult to directly compare prenatal depression levels using different scales, the study of 200 pregnant women in Korea identified 33.5% as having significant depressed mood (EPDS score of ≥10) [18]. This may be due to differences in participant characteristics such as monthly income. In this study, 53.8% had a monthly household income of 4 million KRW or more, which is higher than the previous Korean study (42.5%) [18]. Using the same instrument, however, the depression level (2.35 points) in this study was also lower than in a study conducted in the United States (7.0 points) [26]. Also, using the same cutoff of 10 points and higher, the study on pregnant women in the United States [26] reported a higher proportion (24.9%) of prenatal depression. This difference may be related to this study’s participants being recruited from the community via the probability-proportional phylogenetic extraction method. Furthermore, the United States study reported between 24.9% and 33.2% of participants having concurrent psychological problems such as sleep disorders, anxiety disorders, and post-traumatic stress. This study aligns with prior studies that reported a relatively low level of depression among Korean pregnant women, suggesting that pregnancy itself is perceived as part of a happy life cycle [27], and the fact that pregnant Korean women receive a lot of respect and love from people around them, thus they may perceive pregnancy as a positive process [28]. Nevertheless, depression in pregnant women can cause various health problems, and the proper assessment and management of depression can lead to healthier pregnancy outcomes.

This study identified household monthly income and education level as the sociodemographic characteristics that were associated with depression in pregnant Korean women. This is in line with a study [29] that reported low monthly income being indicative of a high risk of depression throughout pregnancy; another study [30] showed that depression during pregnancy was more frequent in women with a low education level, and that the odds ratio of depression during pregnancy increased among those who are either unemployed or are housewives. Therefore, it is necessary to assess and manage the risk of depression for pregnant women, particularly if they have such vulnerable characteristics. For pregnant women with a low level of education, policy support including health care and service improvement is necessary to avoid prenatal depression.

In addition, breakfast frequency, sleep time, and drinking were identified as health behavioral factors affecting depression in pregnant Korean women. In other words, the depression score decreased significantly with regular breakfast consumption (as ‘more than 5 days’ a week) and with adequate sleep; in the case of binge drinking, which albeit only constituted 8.1% of the sample, the depression score increased significantly. A healthy lifestyle is essential before pregnancy [31], and unhealthy behaviors have been reported to threaten maternal and child health and affect pregnant women [32]. In addition to guidelines for depression screening during pregnancy [33], regular assessment of drinking while pregnant should be reinforced.

A plant-based diet rich in vitamins lowers the risk of prenatal depression [34], while exercise has been shown to also have a positive effect on women’s mental health during pregnancy [35]. In order to promote healthy behaviors including better nutrition, more exercise, improved sleep, and less drinking, the commitment and support of healthcare professionals, along with the preparation and education of social and institutional health behavior guidelines, are necessary in Korea.

Moreover, this study identified health status factors affecting depression; i.e., subjective and oral health status of expectant mothers. This is similar to another study of Korean women which also identified subjective health status and subjective oral health status as variables related to depression during pregnancy [23]. Subjective health status refers to the perception that one’s health status has changed while experiencing physical changes due to pregnancy. Thus, to reduce depression during pregnancy, it is important to have a positive attitude toward one’s health status. In addition, despite the importance of self-evaluation of oral health status, the majority of adults lack awareness of its importance and are prone to neglect this aspect of their physical well-being, which, in turn, negatively affects one’s overall health [36]. As such, a system that continuously monitors the health and oral health of pregnant women is necessary in order to promote well-being during pregnancy and should include education concerning general health promotion and emphasis on basic oral health [23]. Such emphasis on subjective health and subjective oral health should be offered to all women from the early stages of pregnancy.

This study identified stress as a psychological factor affecting prenatal depression, which supports a prior study [9]. Pregnant women’s stressors include not only the pregnancy itself, but underlying issues of poverty, unemployment, economic status, and relationship changes as well [37]. Improving women’s mental health during the prenatal period is important for both mother and child outcomes [18]. It is necessary to systematically consider the stress of pregnant women during prenatal care, and more attention should be paid to identifying psychological risk factors in pregnant women [38]. As such, this study provides further evidence to check and manage the physical condition of pregnant women and fetuses at prenatal checkups, as well as to implement a stress assessment and management program in order to respond more flexibly to everyday stressors.

This study did not find that the age of pregnant women nor their unmet healthcare needs affected depression. According to previous studies, the prevalence of depression during pregnancy has been reported as increasing among younger women in the United Kingdom [14]; in contrast, that age has no effect on prenatal depression [9]. While this study supports the latter report, Korea has a national health insurance system and Korean pregnant women show high rates (95.2%) of early prenatal care (e.g., first visit before 8 weeks into pregnancy) and overall prenatal care (nearly 100%) [39], which may differ from the United Kingdom [14]. Furthermore, 91.8% of pregnant Korean women had reported not having any unmet healthcare needs during pregnancy [31], which matches this study’s findings; As such, this may be considered in its non-relationship with depression.

A limitations of this study was that stress was the only psychological parameter measured during pregnancy in the data available from KCHS. Future studies reflecting a wider spectrum of positive and negative psychological factors throughout pregnancy are thus required. In addition, as the KCHS data did not allow classification of the pregnancy into the first, second, or third trimester, it was impossible to ascertain differences in depression according to pregnancy period. Another limitation is that although prenatal depression is related to obstetrical characteristics (e.g. parity, miscarriage experience, complications), this information was limited due to the nature of a secondary analysis study. Nevertheless, this study offers significant information on the degree of influence on prenatal depression, using probability-proportional sampled data.

In conclusion, this study using a nationally representative database found that low income and low education level, skipping breakfast, less than 8 hours of sleeping, binge drinking, higher stress, and lower subjective health status and subjective oral health status were influential on depression in pregnant Korean women. Findings support the need for healthcare policies and implementing clinical screening to alleviate prenatal depression, especially for pregnant women with low socioeconomic status, poor health behavior, poor health status, and high stress.

## Figures and Tables

**Table 1.** General characteristics of participants and depression by characteristics of participants (N=1,096)

Concept	Variable	Categories	Weighted % or mean±SE	Data range	Possible range	Depression		
						Mean±SE	t or r	*p*
General characteristic	Age (year)		33.9±0.1	19–55			–.01	.741
		19–34	59.9			2.37±0.78	.45	.656
		35–55	40.1			2.32±0.78		
	Monthly household income (KRW)	<4 million	41.3			2.82±0.09	7.13	<.001
		≥4 million	58.7			2.08±0.05		
	Education	Up to high school	22.2			2.40±0.06	2.10	.037
		Junior college graduate or more	77.8			2.16±0.10		
Health behavior	Skipped breakfast	Yes	49.4			2.80±0.09	8.62	<.001
		No	50.6			1.90±0.06		
	Sleep duration (hour/day)	<8	50.2			2.53±0.07	3.40	.001
		≥8	49.8			2.16±0.08		
	Binge drinking	Yes	8.1			2.85±0.11	4.43	<.001
		No	91.9			2.30±0.06		
Health status	Subjective health status		3.58±0.01		1–5		–.26	<.001
	Subjective oral health status		3.13±0.01		1–5		–.20	<.001
	Unmet healthcare needs experiences	Yes	3.9			3.69±0.19	7.10	<.001
		No	96.1			2.27±0.05		
Psychological characteristic	Stress score		1.98±0.01	1–4	1–4		.42	<.001
	Depression score		2.35±0.06	0–23	0–27			
		None	82.9		0–4			
		Mild	12.5		5–9			
		Moderate	3.5		10–14			
		Moderately severe	0.9		15–19			
		Severe	0.2		20–27			

KRW: Korean won (1 million KRW is approximately 900 US dollars).

**Table 2.** Associated factors of depression in pregnancy (N=1,096)

Concept	Variable	B	SE	95% CI	t	*p*
Constant		1.45	0.31		4.73	<.001
General characteristic	Monthly household income^†^	0.69	0.11	0.48–0.90	6.45	<.001
	Education^†^	0.7	0.12	0.48–0.93	5.98	<.001
Health behavior	Skipping breakfast^†^	0.34	0.10	0.15–0.53	3.48	.001
	Sleep duration^†^	0.26	0.10	0.07–0.45	2.68	.009
	Binge drinking^†^	0.46	0.13	0.20–0.73	3.46	.001
Health status	Subjective health status	–0.59	0.07	–0.72 to –0.46	–8.78	<.001
	Subjective oral health status	–0.17	0.06	–0.29 to –0.06	–3.06	.003
	Unmet healthcare needs experiences^†^	0.11	0.16	–0.21–0.43	0.67	.504
Psychologic characteristic	Stress score	1.89	0.06	1.78–2.01	31.7	<.001
	R^2=^.244, Wald F=268.61, *p*＜.001					

† The reference groups were as follows: monthly household income (≥4 million Korean won), education (up to high school), skipping breakfast (no), sleep duration (≥8 hours per day), binge drinking (no), and unmet healthcare needs experiences (no).
